# Methylenetetrahydrofolate reductase and psychiatric diseases

**DOI:** 10.1038/s41398-018-0276-6

**Published:** 2018-11-05

**Authors:** Lin Wan, Yuhong Li, Zhengrong Zhang, Zuoli Sun, Yi He, Rena Li

**Affiliations:** 1 Center for Brain Disorders Research, Capital Medical University & Beijing Institute of Brain Disorders, Beijing, 100069 China; 2 The National Clinical Research Center for Mental Disorders & Beijing Key Laboratory of Mental Disorders of Mental Disorders, Beijing Anding Hospital, Capital Medical University, Beijing, 100088 China; 3 Center for Hormone Advanced Science and Education, Roskamp Institute, Sarasota, FL 34243 USA

## Abstract

Methylenetetrahydrofolate reductase (MTHFR) is a key enzyme for the critical process of one-carbon metabolism involving folate and homocysteine metabolisms. It is known that some polymorphism of *MTHFR* would result in reduction of MTHFR enzyme activity as well as DNA methylation process, later shown to have significant impacts in various psychiatric diseases. However, it is unclear whether the polymorphism of *MTHFR* could be an independent or an add-on risk factor for specific psychiatric symptoms, such as anxiety, depression, positive, or negative symptoms of schizophrenia, or acts as risk factor for specific psychiatric disorders, such as schizophrenia, major depression, autisms, and bipolar disorders. It is also understudied on whether folate supplements could be an effective treatment for psychiatric patients with defect MTHFR activity. In this review, we not only gathered the most recent discoveries on *MTHFR* polymorphism and related DNA methylation in various psychiatric disorders, but also highlighted the potential relationships between MTHFR activity and implication of folate-related function in specific mental diseases.

## Introduction

Methylenetetrahydrofolate reductase (MTHFR) is a key enzyme of folate metabolism in the process of one-carbon metabolism. MTHFR converts 5,10-methylenetetrahydrofolate to 5-methyltetrahydrofolate and participate in folate and homocysteine conversion correlated to DNA methylation^1^. As consequences of polymorphism of *MTHFR*, reduction of MTHFR enzymatic activity would cause impaired methylation as well as deficiency of folate. There are plenty of relevant studies on linkage between MTHFR and human diseases including cardiovascular diseases, tumors, neurologic diseases, and psychiatric disorders^2–5^. Moreover, there are stratified factors that have been identified to be involved in the relationship between MTHFR and diseases, such as gender, age, and ethnicity^6–9^. As both DNA methylation and folate are important in mental health, reduction of MTHFR activity or folate deficiency have been associated with an onset of several psychiatric diseases^10^, schizophrenia, bipolar disorder, depression, autism, and ADHD. In this review, we specifically focus on the *MTHFR* polymorphism and related methylation and folate effects on psychiatric diseases as well as the possibility of relationship between clinical phenotypes of MTHFR-related diseases and effectiveness of clinical treatment in psychiatric patients^11^.

## MTHFR

### *MTHFR* gene

**Fig. 1:**
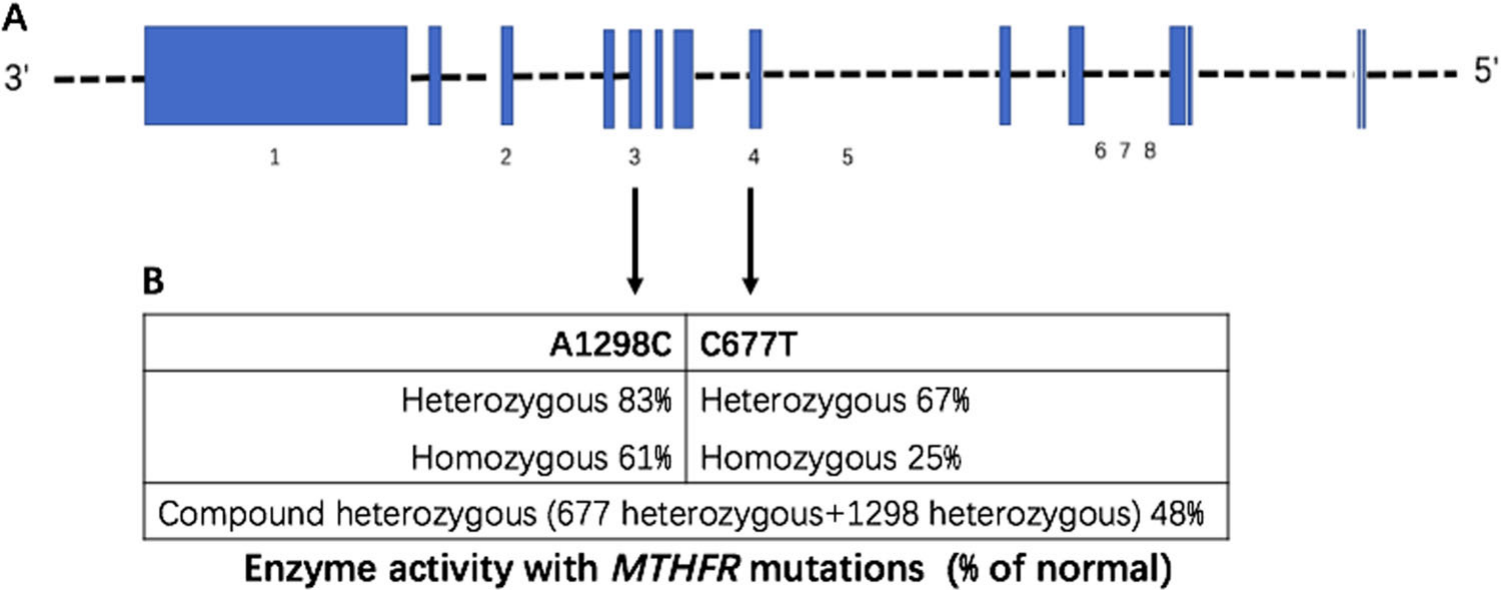
MTHFR enzymatic activity with *MTHFR* mutations. **a** *MTHFR* gene exons are indicated as blue regions, and gene 5’ to 3’ end are from right to left. Numbers under the arrows represent the SNP sequence number, which corresponds to the gene rs-number in the annotation. 1. Rs4846080(A > G); 2. rs1476413(G > A); 3. Rs1801131(A > C); 4. rs1801133(C > T); 5. rs17421511(G > A); 6. rs17037396(C > T); 7.rs9651118(T > C); 8. rs17367504(A > G). **b** Decrease in enzyme Activity (% of normal) with the presence of MTHFR variants

In humans, the *MTHFR* resides on chromosome 1 location p36.3 and was originally described as containing 12 exons as shown in Fig. 1. Human *MTHFR* transcripts are respectively at 2.2 kb, 7.5 kb, and 9.5 kb^12^. The cDNA of 2.2 kb-fragment sequence codes for a 656 residue and 70–77 kDa protein^13^. The cDNA of 7.5 kb and 9.5 kb sequence code a second isoform of 77 kDa protein. Among the exons of *MTHFR*, the first one is noncoding^1^. Apart from the coding region, variable 5’ and 3’ noncoding regions (UTR) were identified, resulting in transcript heterogeneity. The 5’ and 3’ termini of the *MTHFR* cDNA overlap with the 5’ terminus of a chloride ion channel gene and the 3’ terminus of an unidentified gene, respectively. The *MTHFR* gene has multiple promoters and several polyadenylation sites creating 3’UTR lengths of 0.2 kb ± 5.0 kb or 0.6 kb ± 4.0 kb in human^12^. The *MTHFR* gene has been identified to possess 14 common or rare single nucleotide polymorphism that are associated with enzymatic deficiency^14^. Among them rs1801133(C677T) and rs1801131(A1298C) are most reported that may reduce the MTHFR activity in various degrees. For C677T, the enzyme activity of heterozygous and homozygous mutant individuals are respectively 67 and 25% of the wild-type ones. And for A1298C, the enzyme activity of heterozygous and homozygous mutant individuals are respectively 83 and 61% of the wild-type subjects^15^, as shown in Fig. 1.

### MTHFR and its activity

**Fig. 2:**
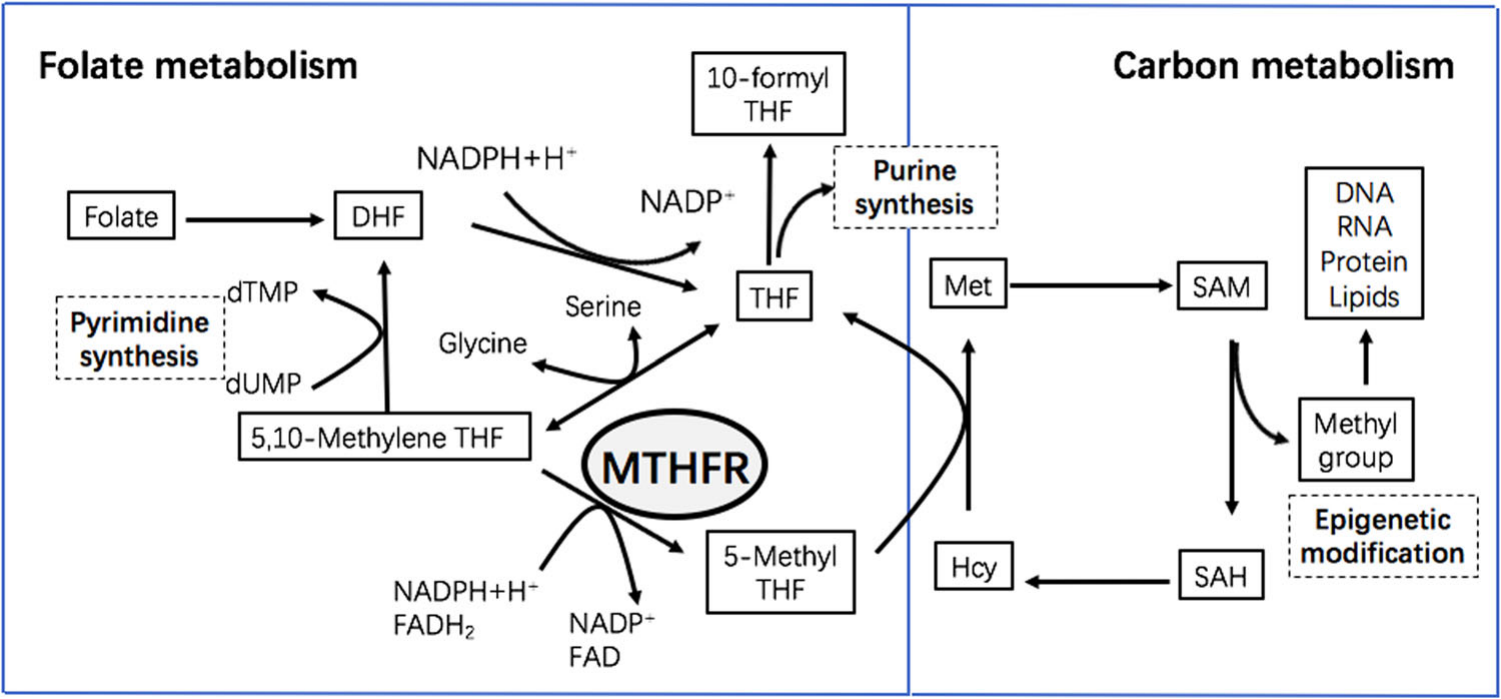
One-carbon metabolism. MTHFR is a key enzyme to catalyze conversion of 5,10-methylene THF to 5-methyl THF and contribute to generation of SAM, which is the direct donor of methyl group. DHF, dihydrofolate acid; THF, tetrahydrofolate acid; MTHFR, methylenetetrahydrofolate reductase; dTMP, deoxythymidine monophosphate; dUMP, deoxyuridine monophosphate; NADPH, nicotinamide adenine dinucleotide phosphate; FAD, flavine adenine dinucleotide; Met, methionine; Hcy, homocysteine; SAM, S-adenosylmethionine; SAH, S-adenosylhomocysteine

**Fig. 3:**
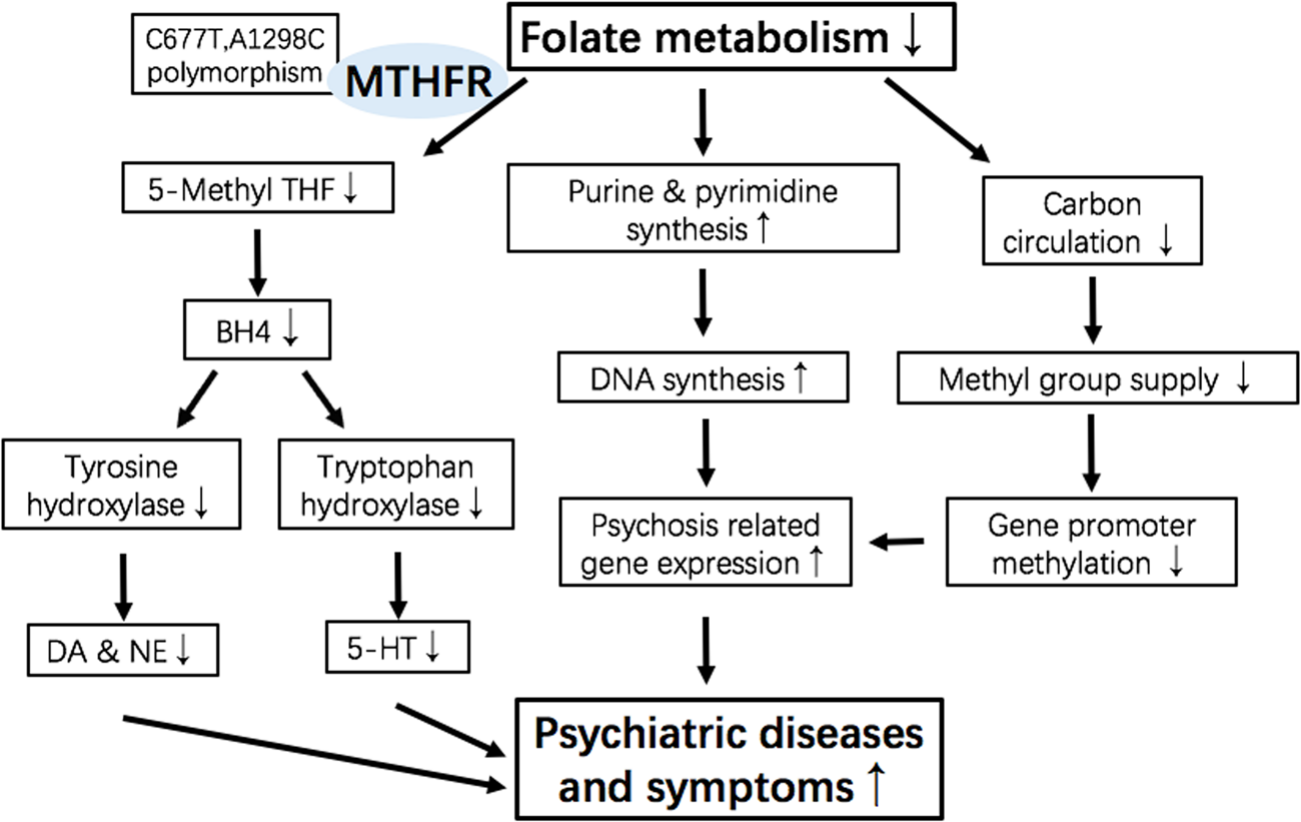
Potential mechanisms of *MTHFR* in psychiatric diseases. Methyl group supply in one-carbon metabolism is affected by *MTHFR* enzyme catalytic process. MTHFR polymorphism affects downstream methylation of schizophrenia-related proteins. DA, glutamate and so on. BH4, tetrahydrobiopterin; DA, dopamine; NE, norepinephrine; 5-HT, 5-hydroxytryptamine

While MTHFR gene codes for different variants, the most common form of MTHFR in human is a 656 amino acids protein. Human MTHFR consists of an N-terminal catalytic domain (amino acids 1–356) which binds 5,10-methylenetetrahydrofolate (5,10-methylene THF), and a C-terminal regulatory domain (amino acids 363–656) which binds S-adenosylmethionine (AdoMet, SAM)^16,17^. As shown in Fig. 2, MTHFR catalyzes the physiologically irreversible reduction of 5,10-methylene THF to 5-methyltetrahydrofolate (5-methyl THF), and plays a critical role in one-carbon metabolism for the reaction of producing methyl groups to participate in epigenetic regulation^18^. The properties and crystal structure of MTHFR from the bacterium Thermus thermophilus HB8 have been determined^19^. While the regulation of MTHFR activity is closely controlled by SAM at C-terminal regulatory domain, more studies indicated that the human MTHFR enzyme activity is also regulated by multiple phosphorylated sites on a serine-rich N-terminal extension region^20^. The phosphorylation leads downregulation of MTHFR activity and upregulation of allosteric inhibition by SAM. It is suggested that phosphorylation impacts on the allosteric regulation of MTHFR via altering the equilibrium of active and inactive states of the enzyme, favoring the inactive state which SAM preferentially binds^21^. The active form of MTHFR could impact on the generation of 5-methyl THF, which is the active form of folate in vivo. Then methionine level increases and related methyl group donation is driven which successively exert potential mechanism on psychiatric diseases, as shown in Fig. 3.

### MTHFR and DNA methylation

Another important role of MTFHR is to participate in donating methyl group to regulate epigenetic modification in the one-carbon metabolism. Methylation is a common regulation process of gene expression that influences cellular development and function^22^, which is dependent on SAM as a methyl donor. SAM originated from methionine cycle in which 5-methyl THF transfers methyl groups to homocysteine in a reaction catalyzed by methionine synthase to produce methionine. In this process, 5,10-methylene THF play a critical role in methionine regeneration and methyl donation, meanwhile MTHFR catalyzes the irreversible conversion of 5,10-methylene THF to 5-methyl THF that participate in generation of SAM in methionine cycle and offer methyl group^23^.

## *MTHFR* polymorphism

### *MTHFR* polymorphisms and enzymatic activity

There are several sites of *MTHFR* polymorphism that have been reported including 2 enzyme activity associated locuses C677T and A1298C and 6 enzyme activity unassociated locuses^6^. As shown in Table 1, with regard to the association of *MTHFR* gene and its enzyme products, some of the studies revealed severe enzymatic deficiency. The encoding of *MTHFR* appears to be polymorphic such as the gene site C677T, one of the most studied and clinically important variant in exon 4. The C677T variant results from a single nucleotide substitution at this position, in which cytosine is replaced by thymine resulting a conversion of alanine to valine residue^24^. The substitution lowers the affinity of MTHFR and its cofactor, which promotes the thermolability and diminishes the enzyme activity. Comparing with wild genotype (CC), the heterozygote (CT) and mutation homozygote (TT) lead to the decline of enzyme activity by about 34 and 75%, and increased thermolability in lymphocyte extracts^25^. In 2001, the Ala222Val mutation was created in human MTHFR, and the mutant protein was successfully purified and its properties were determined. Different from the former studies, the Ala222Val variant exhibits identical catalytic properties as the wild-type enzyme, but it is thermolabile^17^.

**Table 1:** MTHFR and Psychiatric diseases

Gene locus	Diagnosis	Subjects (F/M)	Mean age (F/M)	Genotype number	Allele number	Comments	Country	Year [Ref.]
C677T	SCZ	SCZ 200(99/101)	32.7/9.6	CC 113, CT 68, TT 19	C 294, T 106 C	1.5 and 1.7-fold times higher distribution of T allele in SCZ and BD patients, SCZ patients TT was 2.5 times higher than controls.	Poland	2006^46^
	BPD	BPD 200(95/105)	46.0/43.5	CC 108, CT 73, TT 19	289, T 111 C			
		Controls 300(141/151)	31.0/29.5	CC 210, CT 79, TT 11	499, T 101			
	SCZ	Patients 254(71/183)	41 ± 14	CC 112, CT 111, TT 31	C 335, T 173	TT genotype associated with an increased of schizophrenia. CT compared to CC subjects accounted for an increased of schizophrenia	Netherland	2005^45^
		Controls 414(236/178)	51 ± 14	CC 212, CT 166, TT 36	C 590, T 238			
	SCZ	Patients 200(94/106)	43.4	CC 97, CT82, TT 21	C276, T124	Increased 677 T allele load confers risk for negative symptoms in SCZ	USA	2006^47^
	SCZ	Patients 200(62/138)	43.4	CC 97, CT82, TT 21	C276, T124	TT exhibited significantly greater deficits on VFT, had more difficulty achieving the first category on the WCST and did not differ in CVLT.	USA	2006^48^
	SCZ	Scandinavian820(341/479)		CC 401, CT 342, TT 76	C 1144, T 494	Patients of the C677T significantly affected age at onset of schizophrenia with lower age of onset with increasing numbers of the mutant T allele.	Scandinavian & Chinese	2009^49^
		Chinese 243(120/123)						
	SCZ	Patients 85(60/25)	37 ± 10	CC 47, CT26, TT 12	C 120, T 50	A significant association for *MTHFR* 677TT in the male, and 677CT genotype in the total patients group.	Syria	2012^51^
		Controls 126(47/79)	40 ± 10	CC 58, CT58, TT 10				
	SCZ	SCZ 1002(462/540)	31.2 ± 9.9	CC 160, CT 450, TT 384	C 770, T 1218	677 T allele have effect on risk of schizophrenia, memory impairment, and gray matter density.	China	2013^52^
		Controls 1036(434/602)	32.5 ± 8.3	CC 213, CT 505, TT 318	C 931, T 1141			
	SCZ	SCZ 1149(473/676)	54.6 ± 14.9	CC 417, CT 530, TT 202	C 1364, T 934	a significant association between the *MTHFR*	Japan	2014^53^
		controls 2742(1512/1230)	38.8 ± 12.6	CC1072,CT1260, TT 410	C3404, T 2080	C677T polymorphism and schizophrenia.		
		SCZ 621(319/302)	46.5 ± 15.8	CC 220, CT 309, TT 92	C 749, T 493			
		controls 486(255/231)	35.0 ± 12.7	CC 174, CT 239, TT 73	C 587, T 385			
	SCZ	Cases 143		CC51, CT 70, TT 22	C 172, T 114	A weak haplotype analysis association for the 1298C-677C haplotype.	China	2010^60^
		Controls 235		CC 71, CT 123, TT 41	C 265, T 205			
	SCZ	Cases 90(32/58)	42.91	CC40, CT 37, TT 12	C 117, T 61	*MTHFR* polymorphisms interacted on cognition, and the *MTHFR* T-allele attenuated the cognitive effects.	Greece	2013^97^
		Controls 55(25/30)	43.69	CC 21, CT 22, TT 12	C 64, T 46			
	SCZ	Cases 696				*MTHFR* polymorphisms are not related to the development of schizophrenia strong support for association of C677T with schizophrenia.	Japan	2010^6^
		Controls 747						
	SCZ	Cases 3213				*MTHFR* polymorphisms do not influence age of onset in schizophrenia	East Asia & Caucasia	2010^7^
	SCZ	Cases 742(185/557)	39.0 ± 14	CC 334, CT 322, TT 86	C 990, T 494	Neither winter birth nor *MTHFR* were significantly associated with increased schizophrenia risk	Netherland	2007^8^
		Controls 884(477/407)	52.0 ± 20	CC 405, CT 387, TT 92	C 1197, T 571			
	SCZ	SCZ 103(35/68)	33.9 ± 9.4	CC 52, CT 36, TT 15	C 140, T 66	*MTHFR* C677T polymorphisms are associated with the risk of developing BD and schizophrenia and influence the age at onset of BD but not schizophrenia.	Egypt	2014^62^
		BD 134(62/72)	32.2 ± 10.9	CC 46, CT 70, TT 18	C 162, T 106			
		Controls 149(73/76)	34.3 ± 6.0	CC 114, CT 30, TT 5	C 258, T 40			
	BPD	Cases 501	37.8 ± 12.7	CC 178, CT 231, TT 73	C 587, T 415	*MTHFR* C677T variant was not to play a major role in the susceptibility to bipolar disorder.	China	2009^64^
		Controls 461	36.6 ± 7.2	CC 153, CT 235, TT 92	C 541, T 381			
	BPD	Cases 846(553/293)	47.2 ± 11.9	CC 362, CT 386, TT 98	C 1110, T 582	No association for genotypic or allelic in this sample.	UK	2010^65^
		Controls 1576(906/670)	42.1 ± 13.2	CC 642, CT 719, TT 215	C2003, T 1149			
	SCZ	Cases 66(21/45)	29.0 ± 4.0	CC 35, CT 27, TT 4	C 97, T 35	failed to find interaction between C677T polymorphism and vulnerability to schizophrenia and bipolar disorder.	Iran	2011^66^
	BPD	Cases 90(39/51)	35.0 ± 8.0	CC 52, CT 34, TT 4	C 138, T 42			
		Controls 94(41/53)	31.0 ± 6.0	CC 54, CT 38, TT 2	C 146, T 42			
	DD	Cases 100(63/37)	47.7 (18–83)	CC 30, CT 56, TT 14	C 116, T 84	C677T genotype associated with increased risk of depressive episodes in this study.	UK	2004^67^
		Controls 89(51/38)	51.2 (25–84)	CC 40, CT 37, TT 12	C 117, T 61			
	MDD	Cases 147(103/44)	47.4 ± 11.3	CC 63, CT 68, TT 16	C 194, T 100	677CC genotype showing the most severe symptom severity course over the 60 months of observation.	Australia	2013^68^
	MDD	Cases 368(278/90)	51.54 ± 16.40	CC 88, CT 259, TT 21	C 435, T 301	The T allele and C/T genotype of C677T were different between cases and controls.	China	2014^75^
		Controls 219(139/80)	44.42 ± 16.52	CC 113, CT 91, TT 15	C 317, T 121			
	Anxiety	Cases 621(431/190)		CC 308, CT 263, TT 50	C 879, T 363	TT genotype was significantly related to depression without comorbid anxiety and no significant association to anxiety.	Norway	2003^69^
	DD	Cases 242(100/142)		CC 127, CT 85, TT 30	C 339, T 145			
	DD (postmenopausal)	Cases 83	54.2 ± 4.7 (cases + controls)	CC 26, CT 38, TT 19	C 90, T 76	TT genotype displayed a 4.831-fold increased risk of moderate and severe depression.	Poland	2008^70^
		Controls 89		CC 46, CT 36, TT 7	C 128, T 50			
	DD	Pregnancies 6809	28.3 ± 4.71	CC3035, CT3017, TT757	C9087, T4531	Pregnancies folic acid supplements protected against depression, more obvious in TT genotype.	UK	2011^98^
	DD	cases 4992				*MTHFR* C677T polymorphism contributed to the increased depression risk in overall populations	East Asia & Caucasia	2013^73^
		controls 17082 in 26 studies						
	DD in TCEs	Cases 124(92/32)	44.5	CC 60, CT 50, TT 14	C 170, T 78	T-allele carriers may be at an increased risk for MDD recurrence after exposure to TCEs.	Netherland	2013^71^
		Controls 665(372/293)	20.5	CC 306, CT 239, TT 20	C 751, T 279			
	DD	NAME 1017(768/249)	75.3	CC + CT 906, TT 111		did not find an association between the TT genotype and impaired cognition or depression.	USA	2012^76^
		BPRHS 939(674/265)	57.9	CC + CT 823, TT 116				
	DD	Cases 82		CC 31, CT 34, TT 17	C 96, T 68	No significant differences were found in frequency of the T allele or the *MTHFR* C667T TT genotype between the depressed and controls.	USA	2011^77^
		Controls 74		CC 33, CT 28, TT 13	C 94, T 54			
	DLD&Anxiety	Cases 240	74.7 ± 4.4	CC 98, CT 113, TT 29	C 309, T 171	C677T gene variation does not play a role in the modulation of mood and cognitive performance.	Australia	2005^78^
	MDD	Cases 1222(841/381)	47.2 ± 12.0 (46.59 ± 12.31/48.59 ± 11.71)	CC 545, CT 513, TT 164	C 1603, T 841	no significant differences in C677T or T allele frequencies between DD patients and controls.	UK	2008^74^
		Controls 835(464/371)	49.1 ± 8.1 (47.31 ± 9.23/48.47 ± 6.92)	CC 350, CT 379, TT 106	C 1079, T 591			
	ASD	Cases 39(8/31)	8.83 ± 0.84	CC 21, CT 14, TT 4	C 56, T 22	a normal distribution of polymorphism in ASDs, but the frequency of T allele was more prevalent.	Romania	2009^80^
		Controls 43(14/29)	9.05 ± 0.91	CC 25, CT 15, TT 3	C 65, T 21			
	ASD	Cases 147(40/107)	7.9 ± 4.5	CC 65, CT 62, TT 20	C 192, T 102	four behaviors were more common and at least one copy of T allele as compared to homozygous wildtype individuals. No differences existed among genotypes for level of functioning	USA	2009^81^
	ASD	ASD 429(57/372)				Periconceptional folic acid may reduce ASD risk in those with inefficient folate metabolism.	USA	2012^99^
		DD 130(44/86)						
		TD 278(50/228)						
	ASD	Cases186(48/138)	8.1 ± 4.3	CC 79, CT 77, TT 30	C 235, T 137	The TT frequency in children with autism was significantly higher than those in controls.	China	2012^83^
		Controls186(45/141)	8.2 ± 4.1	CC 87, CT 83, TT 16	C 257, T 115			
	ASD	Cases 249(24/225)		CC 76, CT 136, TT 37	C 288, T 210	677CT/1298AC was significantly associated with an risk of ASD by 2.11-fold to 677CC/1298AA in males but not females	Korea	2014^84^
		Controls 423(169/254)		CC 139, CT 204, TT 80	C 482, T 364			
	ASD	Cases151(35/116)		CC 60, CT 68, TT 23	C 188, T 114	The genotypes did not show differences between cases and controls, nor association between the T allele and selected behaviors.	Brazil	2010^100^
		Controls100(43/57)		CC 45, CT 41, TT 14	C 131, T 69			
	ASD	Cases 98(27/71)	6.0 ± 2.1	CC 44, CT 51, TT 3	C 139, T 57	677T-allele frequency was higher in autistic children compared with controls, not signicantly.	Turkey	2014^101^
		Controls 70(24/46)	5.0 ± 1.0	CC 37, CT 33, TT 0	C 107, T 33			
	ADHD	Cases 48(16/32)	4.1 ± 4.2	CC 23, CT + TT 25		a 1.3-fold increase for C677T locus predominant linkage to the inattentive symptoms.	USA	2008^85^
	ADHD	Cases 40(9/31)	9.77 ± 2.3	CC 22, CT + TT 18		no significant differences in genotype distributions of the C677T alleles between ADHD and controls.	Turkey	2011^86^
		Controls 30(7/23)	10.5 ± 4.5	CC 15, CT + TT 15				
	ADHD	Cases 580(52/528)				the folate–homocysteine pathway gene variants may affect ADHD through mild hyperhomocysteinemia and vitamin B12 deficiency.	India	2017^102^
		Controls 286(156/130)						
	ADHD	Cases100(20/80)	8.87 ± 2.55	CC 44, CT 47, TT 9	C 135, T 65	did not find any association between *MTHFR* 677 T allele, *MTHFR* 1298 C allele, and ADHD.	Turkey	2012^87^
		Controls 300(60/240)	8.02 ± 2.69	CC 154, CT 125, TT 21	C 433, T 167			
A1298C	SCZ	Cases 200(94/106)	43.4	AA 99, AC83, CC 18	A281, T119	No significant role for the A1298C polymorphism in schizophrenia symptoms.	USA	2006^47^
	SCZ	Cases 379(159/220)	32.1 ± 9.7	AA230, AC127, CC22	A587, C171	an association between the 1298C allele and SCZ	China	2010^59^
		Controls 380(165/215)	31.5 ± 8.6	AA260, AC108, CC12	A628, C132			
	SCZ	Cases 143		AA88, AC49, CC6	A225, C61	maternal *MTHFR* 1298C allele associated with a significantly increased risk of schizophrenia.	China	2010^60^
		Controls 235		AA171, AC61, CC3	A403, C67			
	MDD	Cases 147(103/44)	47.4 ± 11.3	AA69, AC63, CC15	A201, T93	No association between A1298C and MDD	Australia	2013^68^
	ASD	Cases 249(24/225)		AA 147, AC 75, CC 14	A369, C103	significant associations between autistic disorder or atypical autism and 1298AC polymorphism	Korea	2014^84^
		Controls 423(169/254)		AA 298, AC 114, CC 11	A710, C136			
	ADHD	Cases 48(16/32)	4.1 ± 4.2	AA 25, AC +CC 23		A1298C was predominant linkage to inattentive symptoms, a 7.4-fold increase in diagnosis.	USA	2008^85^
	ADHD	Cases 40(9/31)	9.77 ± 2.3	AA 9, AC +CC 31		A1298C alleles was different between the ADHD patients and the controls.	Turkey	2011^86^
		Controls 30(7/23)	10.5 ± 4.5	AA 14, AC +CC 16				

*SCZ* schizophrenia, *BPD* bipolar Disorder, *DD* depression disorder, *MDD* major depression disorder, *NAME* the nutrition, aging, and memory in elders, *BPRHS* the Boston Puerto Rican Health Study, *ASD* Autism spectrum disorders, *ADHD* attention deficit hyperactivity disorder, *MD* mood disorder, *TCEs* traumatic childhood events, *DLD* development delay, *TD* typical development, *AD* Alzheimer disease, *MCI* mild cognition impairment

Another common polymorphism is A1298C, in which adenine is replaced by cytosine resulting a conversion of glutamate to alanine at 429 residue, which also diminishes the enzyme activity. Lymphocyte extracts from homozygous 1298CC individuals showed 61% of wild-type enzyme activity^26^. The Ala177Val was established in the MTHFR of *E. coli* to study the biochemical phenotype of the Ala222Val variant. Then literatures reported the Ala177Val mutation has no influence on the kinetic parameters of bacterial MTHFR, but rather reduces enzyme stability and affinity for cofactor, and thus increases the tendency to form inactive enzyme via flavin dissociation, compared to the wild-type enzyme^27^.

### *MTHFR* polymorphism and methylation

*MTHFR* polymorphism is also associated with global methylation activity. For example, a study of coronary artery patients indicated that genomic DNA methylation directly correlates with folate status and inversely with plasma homocysteine levels. After genotype analysis, TT genotypes had a diminished level of global DNA methylation compared with those with CC wild type^28^. Such a change was also found in healthy individuals which showed reduction of DNA methylation in individuals with the TT *MTHFR* genotype compared to subjects with CC *MTHFR*^29^. While DNA methylation may be age, gender, and cell-type specific, *MTHFR* polymorphism might not be always associated with hypomethylation of DNA. For example, a study of aging-related DNA methylation found hypomethylation in aged individuals compared to young populations without significant association with C677T *MTHFR* genotypes^30^. Studies also demonstrated no significant inference of *MTHFR* C677T polymorphism in global DNA methylation in oral epithelial cell samples^31^ or lymphocytes of healthy individuals^32^, as well as cutaneous squamous cell carcinoma in renal transplant patients^33^. Those reports suggested a *MTHFR* polymorphism independent mechanism in aging and cell-type specific global DNA methylation. Furthermore, a similar results were reported in a study of individuals with or without oligozoospermic which showed no significant association between DNA methylation in spermatozoa and the *MTHFR* C677T genotypes although a trend for higher incidence of methylation alterations in severe oligozoospermic infertile men with CT genotypes were observed^34^, suggesting that a much more complicated or indirect interactions between *MTHFR* polymorphism and methylation are involved.

As global DNA methylation refers to the average methylation status that occurs across the whole genome, *MTHFR* polymorphism could also destruct gene-specific methylation process which refers the methylation status of specific genes. For example, a study of *MTHFR* polymorphism genotypes in colorectal cancer patients reported that the frequency of methylated *Bcl-2* promoter was significantly higher in individuals with CC genotype than that of those with CT and TT genotypes, and a significant difference of C and T alleles distribution were observed between patients with methylated and unmethylated *Bcl-2* promoter^35^. Furthermore, studies of *IGF-2* gene in transitional cell carcinoma of the bladder and *MGMT* gene in gastric cancer showed that patients with CT or TT *MTHFR* genotypes had reduced methylation of *IGF-2* or *MGMT* compared those with CC genotype^36,37^. Together, as *MTHFR* is an important enzyme for folate metabolism which plays critical role in epigenetic as DNA methylation, accumulated evidence showed that global DNA methylation can be associated with *MTHFR* polymorphism genotypes in both healthy populations and individuals with various diseases. However, some cell type- and age-related global DNA methylation showed independent of *MTHFR* genotypes. While the underlying mechanism of *MTHFR* independent global DNA methylation remains unknown, the *MTHFR* polymorphisms related gene-specific DNA methylations were commonly reported in various pathological conditions.

### Mouse models of MTHFR deficiency

The *Mthfr* of mice were knockout to investigate MTHFR deficient by animal models^38^. The Mthfr^+ /−^ mice showed normal growth and similar survival to that of wild-type mice^39^. The Mthfr^−/−^ mice were with none MTHFR enzyme activity in all tissues, whereas the Mthfr^+/−^ showed 60% residual activity, similar to the value observed in patients homozygous for the C667T polymorphism^40^. In the *Mthfr*^+/−^ and *Mthfr*^−/−^ mice, the plasma total homocysteine levels were 1.6- and 10-fold higher, respectively, than the wildtype controls. SAM levels were decreased, but S-adenosylhomocysteine (AdoHcy, SAH) levels were elevated considerably, with global DNA hypomethylation observed in both heterozygotes and homozygotes^38^. Then researchers proposed that heterozygous knockout mice appeared to be a good animal model for individuals homozygous for the C667T polymorphism, whereas the homozygous null mice were a better one for severely MTHFR-deficient individuals^19^. Apart from human studies, mice with heterozygous and homozygous mutation in *Mthfr* C677T still accompany with global DNA hypomethylation, decreased SAM and increased SAH levels^41^.

## *MTHFR* polymorphism and psychiatric diseases

Extensive clinical studies demonstrated a significant linkage between *MTHFR* polymorphism and various diseases, such as cardiovascular diseases, neuronal developmental diseases, cancers as well as psychiatric disorders. Among which, C677T and A1298C polymorphisms of MTHFR have been studied the most in psychiatric diseases and showed significant association with reduction of MTHFR enzymatic activity and methylation. In this session, we will focus on the polymorphisms in the gene encoding for MTHFR in schizophrenia (SZ), bipolar disorder (BPD), depression, autism disorder (ASD) and attention deficit hyperactivity disorder (ADHD). Table 1. summarizes studies including MTHFR polymorphism and psychiatric diseases involved in this review.

### Schizophrenia

For decades ago, there was a report of MTHFR enzymatic activity reduction in two schizophrenia patients which were 18 and 21% percent of the normal level, respectively, while homocysteine remethylation was also defected^42^. Later, a regression model was created in a study of *MTHFR* C677T genotype and DNA methylation in schizophrenia subjects, which found females with TT genotype were associated with the lowest global methylation^43^.

Amounts of studies have demonstrated that the level of *MTHFR* polymorphism in C677T locus is associated with the risk of schizophrenia. As indicated in a meta-analysis of *MTHFR* consisted of 7 studies, individuals carried with TT homozygotes had the greatest risk of schizophrenia, compared to the subjects with CC wild type and CT heterozygous genotypes^44^. An allele study with well-defined patients and healthy controls indicated that people with CT heterozygotes had the higher risk of schizophrenia than CC carriers^45^. Furthermore, a genotype study also reported that homozygous TT genotype of *MTHFR* was also associated with risk of schizophrenic patients accompanying with bipolar disorder^46^.

It is interesting to mention that the C677T polymorphisms of *MTHFR* also has an influence on symptoms of schizophrenia. For example, an increased T allele load is linked to the increase severity of negative symptoms in schizophrenia, while reducing severity of positive symptoms were also noticed. However, the effect of T allele on the negative symptoms of schizophrenia could be further enhanced by folate deficiency^47^. Furthermore, comparing with CC and CT, schizophrenia patients with TT genotype exhibited greater deficits on the verbal fluency test (VFT) and more difficulties on the Wisconsin Card Sorting Test (WCST), but not in California Verbal Learning Test (CVLT) performance^48^. However, the effect of C677T polymorphisms of *MTHFR* on cognitive function was not significant in normal subjects as a longitudinal cognitive study showed that the *MTHFR* C677T polymorphism was not associated with cognitive performance at baseline or over 12 years^49^. In addition, studies also demonstrated that the C677T polymorphism of *MTHFR* is associated with onset age of schizophrenia in a dose-dependent manner, such as increasing numbers of the mutant T allele is linked with early onset^50^.

The relationship between *MTHFR* polymorphism and schizophrenia in different ethnic population were also investigated. Study of schizophrenic patients and healthy controls in the Arab population from Syria found a strong association between C677T and schizophrenia, which showed higher variant T allele frequency in the patients group. Interestingly, a statistically significant association was found for 677TT genotype under the recessive model in the male patients subgroup, and CT genotype under the overdominant model in the total patients group^51^. Studies of Chinese Han population indicated that the T allele shown associated with schizophrenia as a risk allele^52^ while a case–control association between the *MTHFR* C677T polymorphism and schizophrenia in a Japanese subjects research also demonstrated a strong linkage between the *MTHFR* C677T polymorphism and schizophrenia^53^. Furthermore, a meta-analysis including 38 studies with schizophrenia cases and controls showed the association between C677T polymorphism and risk of schizophrenia in all three ethnic populations—African, Asian, and Caucasian^54^.

Studies of sex differences in *MTHFR* polymorphism might provide some insights for the divergent results from various studies of psychiatric disorders. A strong association between 677T allele and male patients with schizophrenia compared female patients suggest that 677T allele might represent different liability in genders^46^. While little is known on the sex differences in *MTHFR* polymorphisms, sex hormones, such as estrogen is known to play a protective effect in female patients with schizophrenia as for the impact of neurodevelopment and social maturation^55^. On the other hand, testosterone may increase male vulnerability to an adverse illness course compared to estrogen^56^, attributed to its narrower and sometimes unfavorable neuroprotection and neurotransmitter modulation profile^57^. Furthermore, progesterone is reported to benefit neurocognition though enhancement of dopamine release in human males and may also have relevance in male physical and mental health while enhancing the benefits of estrogen through potentiation of estrogen-primed effects on dopamine receptors in male schizophrenic patients^58^.

Except for the C677T, there is another site of *MTHFR* polymorphisms associated with psychiatric disorders. A study with patients of schizophrenia and control subjects showed an association between the A1298C allele and schizophrenia^59^. Another research including 111 families, demonstrated that deficient MTHFR enzyme activity in pregnant women was related to the A1298C variant, which was associated with a higher risk of schizophrenia in the offsprings^60^.

Studies of individual with both SNPs (C677T and A1298C) showed that subjects with heterozygosity for both mutations resulted in an even lower MTHFR activity than heterozygosity for single *MTHFR* mutations, while no subjects carry both homozygote for MTHFR mutations regardless which SNPs^15^ Furthermore, There were studies of multiple polymorphisms of one-carbon metabolism and schizophrenia symptoms showed an increase negative symptoms severity with increase of risk alleles, suggesting a cumulative effects of risk SNPs in one-carbon metabolism^61^.

### Bipolar disorder

In addition to schizophrenia, study demonstrated an association between homozygous 677TT genotype of *MTHFR* gene and bipolar disorder with stronger linkage in male patients than female patients^46^. Another study found a higher prevalence of C677T polymorphism in BD patients than healthy subjects, while patients with BD with early onset carried one copy of the T allele^62^. A meta-analysis of 56 studies examining *MTHFR* C677T in patients and control subjects indicated that the T allele and TT genotype carriers showed significant increased risk of major psychiatric disorders including schizophrenia and bipolar disorder^63^. At the same time, some studies found disparate results. For instance, a study reported no significant association between C677T and bipolar disorder^64^, while another study found no evidence for C677T genotypic or allelic association with BD regardless of type I or II^65^. A study with bipolar patients and schizophrenia subjects also observed no robust differences between patients and controls either for allele frequencies or genotype distribution of C677T polymorphism^66^. These discrepancies may result from population stratifications, explicitly, socio-economic status. On the other hand, the included sample size may play a critical role in divergent results.

### Depression

Depression is another major psychiatric disease. *MTHFR* polymorphism is also noticed in patients with depression. Studies found that *MTHFR* polymorphisms might be related to the episode and prognosis of depressive disorder, not the stage of the disease. For example,a cohort study of depressive patients and healthy controls found that *MTHFR* polymorphism were more common in the individuals with depression history compared to controls^67^, while a study over a 60-month follow-up with depressed subjects indicated that the CC genotype of MTHFR C677T were more likely to have more severe symptoms compared to TT genotype carriers^68^. Another study showed that hyperhomocysteinemia and TT *MTHFR* genotype were significantly related to depression only, not comorbid anxiety disorder^69^. More studies reported that *MTHFR* C677T is associated with risk of depression, such as postmenopausal depression^70^ and childhood trauma related major depression disorder (MDD)^71^. It is important to point out the interaction between *MTHFR* polymorphisms and environmental risks for MDD, such as dietary and stress. For example, a study of inter-relationship between *MTHFR* polymorphism and MDD found that the minor T-allele of MTHFR C677T was associated with increased folate deficiency-related body mass index and homocysteine levels in MDD patients only^72^. Another stress-related MTHFR polymorphism in MDD study showed that traumatic stress in childhood could increase risk of MDD recurrence as well as the development of more severe depressive symptoms in MTHFR TT genotype carriers. This study suggests that the increase of mutant allele number of T in C677T locus will enhance stress risk for depression^71^. Both above studies suggest that MTHFR polymorphisms might enhance the environmental risks (low folate intake, traumatic stress at childhood) for MDD via the interaction between genetic and environmental factors. Such a risk was confirmed by a meta-analysis recruiting 26 published studies which showed an association between *MTHFR* C677T polymorphism and increased risk of depression^73^. However, some studies showed no association between MTHFR and MDD or antidepressant treatment response^74,75^.

Similarly, diverse situation existed in other researches as a study did not find evidence of an association between the *MTHFR* TT genotype and depression in a depression cohort^76^. Another study including depressed subjects indicated no significant differences in frequency of the T allele or TT genotype between the depressed and healthy controls^77^. A research of TT genotype and depression scores revealed that the C677T gene variation does not play an important role in the depression scores^78^. In a meta-analysis, no significant differences in genotype or allele frequencies between depressive patients and controls were observed^74^.

A possible reason for divergent consequences is population stratification as the frequency of the T allele is subject to considerable ethnic and geographic variation^74^. Another possibility is that there is an association of this SNP with another disease that is highly correlated with depression. Indeed it has been hypothesized that depression and vascular disease may be different manifestations of the same genetic substrates^79^. Both of these conditions are a result of the interaction of multiple genetic factors and environment, involving multiple genes with small interactive and additive effects.

### Autism disorder

Comparing to Schizophrenia and depression, relatively limited studies of MTHFR in autism have been conducted. Some studies showed higher frequency of C677T polymorphism in children with ASD than in healthy controls^80^, or associated with ASD behavior phenotypes^81^. A risk study of ASD with typical development indicated significant interaction effects between maternal TT genotype and greater risk for ASD^82^, suggesting MTHFR polymorphism might involve the early development of ASD. Other studies in the Chinese Han and Korean population also found that *MTHFR* C677T and A1298C mutation genes were risk factors for autism in Chinese Han children and Korean population, respectively^83,84^.

### Attention deficit hyperactivity disorder (ADHD)

In terms of the relationship between MTHFR and ADHA, only very few studies have been reported, even with controversial findings. For example, studies demonstrated that A1298C genotype appeared to be the predominant linkage to the inattentive symptoms, leading to a 7.4-fold increase in ADHD, compared with a 1.3-fold increase for the C677T genotype^85^, individuals with ADHD seem to be related to A1298C polymorphisms^86^. However, a research with ADHD and healthy controls reported no association between C677T or A1298C polymorphism and ADHD in Turkish children^87^. Further studies with large sample size or better controls are needed.

In conclusion, *MTHFR* polymorphism not only increase risks for diabetes, cardiovascular diseases, and various cancers, but also increase the risk for various psychiatric diseases. For example, as we described above that MTHFR polymorphism is associated with early onset of schizophrenia and the severity of depressive symptoms in MDD. This is important since neurotransmitter imbalances hypotheses are still the main streams for schizophrenia and MDD. Understanding alternative mechanisms of psychiatric diseases will not only provide potential biomarkers for specific psychiatric diseases, but also new targets for antipsychotic drug development. Due to significant controversial findings in *MTHFR* mutation and DNA methylation in both healthy populations and psychiatric patients, investigation of MTHFR activity in peripheral samples might be important. As yet, the relationships between enzymatic activity and mutation of MTHFR have been reported in general healthy and mental retardation populations as well as in animals, no studies have been found in clinical test of MTHFR activity in psychiatric patients^88–90^. In addition, there are still some shortages on MTHFR mutation and psychiatric disease studies. Except for C677T and A1298C, there were little studies on other SNPs as well as the effect of multiple SNPs on the diseases which may also affect MTHFR activity.

### Clinical treatment strategy for MTHFR-related psychiatric disorders

As MTHFR plays a critical role in one-carbon metabolism, which is composed of folate, homocysteine, vitamin B12, and methylation of DNA, mutation of specific gene locus on *MTHFR* and correlative enzyme activity decline will affect various of physiological events as well as some pathology states, including psychiatric disorders. Whether we could cope with gene mutation and enzyme activity damage using folate one-carbon metabolism strategy as clinical treatment for MTHFR-related psychiatric disease? Some studies showed some interesting possibilities. For example, studies of healthy females found that the low level of serum folate in 677TT genotype is associated with an increase in homocysteine concentration and DNA hypomethylation^91,92^, which reveals the association between *MTHFR* C677T polymorphisms and nutrient status. As food is a major resource for folate, studies reported that low folate level due to unbalanced diet is associated with higher prevalence on schizophrenia, particularly in infants with maternal nutritional deficiency^11,93^. Another study exploring the association between folate and symptoms of schizophrenia indicated that low folate was associated with negative symptoms severity in schizophrenia subjects^94^. One possible role of folate in mental health is its action on DNA methylation and gene expression which have been wildly reported in human psychiatric disorders.

As *MTHFR* polymorphisms-induced MTHFR activity decline is irreversible, clinicians tried to use supplement of folate to help methylation process and change the pathogenesis state. For instance, methylfolate supplement was used for the improvement of psychiatric symptoms^95^, while folate supplementation showed reduction of the incidence of neural tube defects which reduces the incidence of schizophrenia^96^. Although there is no evidence that supplements are helpful in the treatment of psychosis in general, based on the published studies, we believe that if we can detect *MTHFR* polymorphism in individuals with various psychiatric diseases, we might be able to differentiate those MTHFR-related psychiatric patients from non MTHFR deficient patients and develop specific clinical treatment strategies, such as folate or methylfolate supplement to reverse the symptoms. In summary, due to the higher frequency of *MTHFR* polymorphism in various psychiatric disease, supplement of folate and cobalamin might be critical when patients with MTHFR deficiency. MTHFR deficiency-related psychiatric diseases should be identified and might be able to be treated with targeted supplement for the diseases and related symptoms.

## Conclusions

Increasing evidence demonstrated that *MTHFR* polymorphism including C677T and A1298C is associated with psychiatric diseases. The *MTHFR* gene polymorphism is linked to onset, clinical symptoms, prevalence as well as response to treatments. The influence of *MTHFR* on psychiatric diseases is mainly through reduction of MTHFR activity which results in elevation of homocysteine, reduction of DNA methylation-dependent methyl donor, finally induces hypomethylation, and then active disease-related genes. However, some age- and cell type-specific methylation seems independent from *MTHFR* polymorphism. *MTHFR* mutation also can increase environmental risks for psychiatric disorders, such as MDD through interaction between genetic and epigenetic factors. Investigation of MTHFR in psychiatric diseases has important clinical implications, such as identification role of *MTHFR* and its genotypes in the psychiatric patients who respond or not respond to traditional pharmacological treatment for personalized treatment management of psychiatric diseases.
